# Orbital radiotherapy for thyroid eye disease: case report with a review of the literature

**DOI:** 10.1259/bjrcr.20150385

**Published:** 2016-05-28

**Authors:** Andrew Luu

**Affiliations:** Torbay Hospital, Torbay & South Devon NHS Foundation Trust, Torquay, UK

## Abstract

Thyroid eye disease (TED) is a condition mainly associated with Graves' disease. We report a patient with moderate-tosevere TED who underwent a combination of intravenous and oral steroids and orbital radiotherapy that improved her visual acuity and colour vision drastically, and decreased upper lid oedema. We review the clinical features of this unusual condition, our patient's history and treatment, and the knowledge base from the current literature.

Thyroid eye disease (TED) is seen most commonly with Graves’ disease (prevalence of 25–50%).^1^ Symptoms include diplopia, eyelid erythema and retraction, and soreness and grittiness of eyes with increased watering. Severe cases (3–5%) may present with pain, corneal ulceration, optic nerve compression and partial/total loss of vision.^1,2^ Corticosteroids are the primary treatment for active phase TED, although there is risk of adverse effects such as weight gain, hypertension and immunosuppression with long-term, high-dose use.^1–3^ Orbital radiotherapy (OR) is often an adjunct with steroids; this combination has provided a better response than either alone.^3–5^ We discuss a patient’s history with TED before, during and after treatment with OR and steroids, and also opinions concerning this treatment from the literature.

## Case presentation

A 76-year-old Caucasian, non-smoking female suffered a minor stroke in August 2014 owing to hyperthyroidism-induced atrial fibrillation that was later diagnosed as Graves’ disease. She was initially prescribed carbimazole 10 mg to stabilize the disease, which was then increased to 20 mg in January 2015. Thyroxine 25 mcg was initially given to normalize free T4 hormone and hyroid-stimulating hormone, which was then increased to 100 mcg in August 2015.

**Figure 1.**
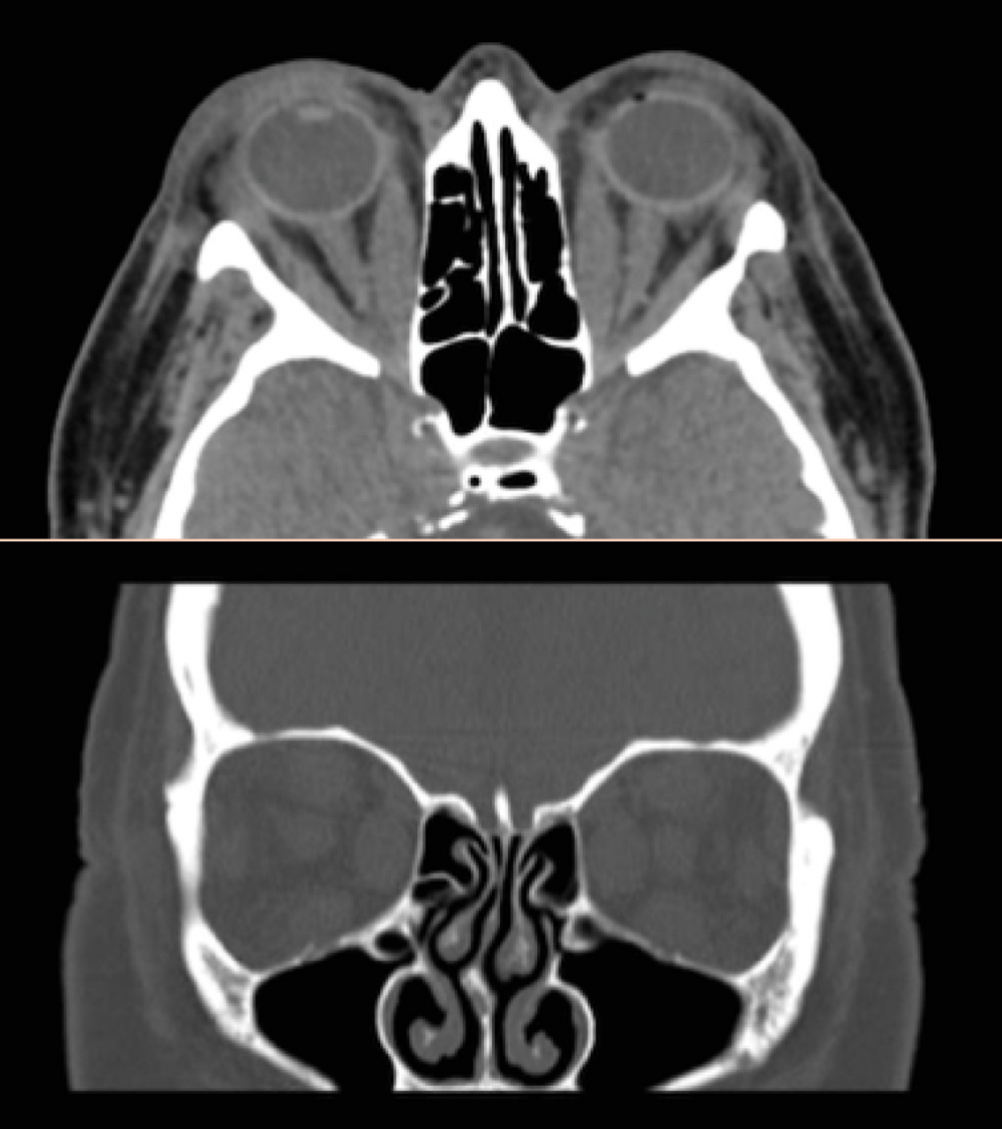
Transverse (upper) and coronal (lower) CT scan of the orbits with intravenous contrast showing expansion of the superior, medial and posterior recti in both orbits with significant bilateral proptosis.

She presented with bilateral upper lid oedema, watery eyes and conjunctival injection in September 2014. This progressed to periorbital but painless oedema and transient diplopia over 6 months. Selenium 200 mcg was also recommended, as it has shown some efficacy in mild TED.^6^ She was given Hypromellose, Viscotears**®**, and Lacri-Lube**®** to maintain moisture and comfort. In March 2015, she regressed with monochromatic vision in her left eye, faded vision in both eyes, significant bilateral proptosis and restricted eye movement. A CT scan of the orbits with intravenous (IV) contrast showed enlarged extraocular muscles in both eyes with possible compression of the left optic nerve (Figure 1). She began steroid therapy with IV methylprednisolone: 500 mg three times for the first week, 250 mg per week for the next 6 weeks and then tapering oral prednisolone starting at 60 mg. She was given alendronic acid to prevent worsening of her osteoarthritis and was also started on omazeprole. There was improvement in left optic nerve function and reduced oedema.

**Figure 2.**
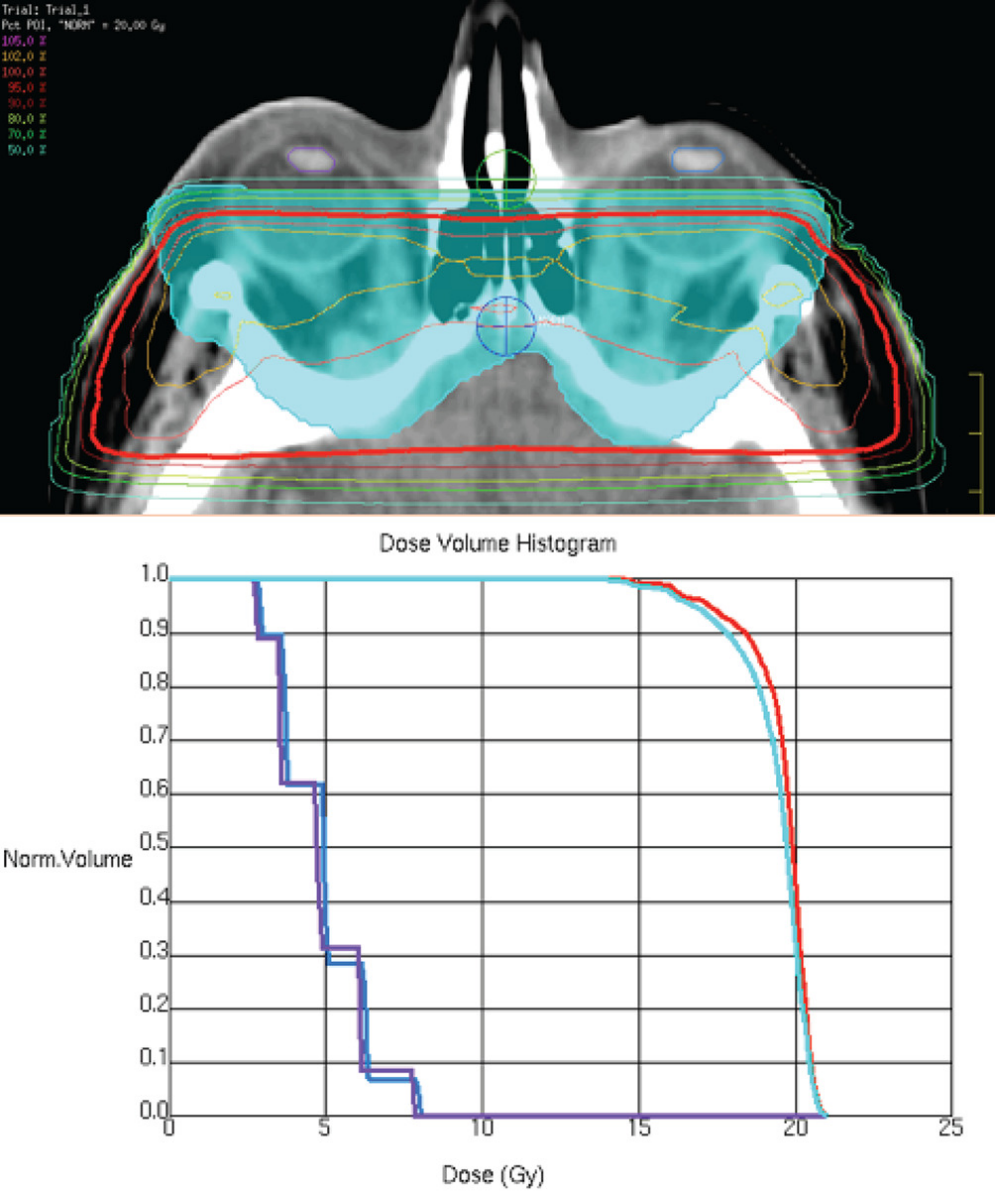
Dose distribution in the axial plane with 95% isodose (red) and PTV in cyan (upper). Dose-volume histogram (lower) with 95% isodose, PTV, left lens (blue) and right lens (purple). PTV, planning target volume.

She commenced OR in May 2015. She was given 20 Gy in 10 fractions over 2 weeks with 6 MV photons, using lateral opposing beams while avoiding the lenses. A Perspex**®** shell immobilized the head and neck. Figure 2 shows the treatment plan and dose volume histogram. She experienced increased inflammation and erythema of the eyelids after the first three treatments, which was managed with steroids and eye drops.

A follow-up in May showed her to be euthyroid with free T4 of 12.6 and her condition was active but stable. She showed overall improvement in visual acuity in both eyes and colour vision in her left eye a month after OR. Another follow-up in August 2015 showed no signs of optic neuropathy, reduction in upper lid oedema, normal pupillary reactions and normal conjunctivae. Her condition was deemed inactive in October 2015, although with some lingering strabismus. This will be corrected by surgery if the disease remains stable after 4 months.

TED affects females five times more than males, and smokers have a five- to seven-fold increased risk.^1,3^ Thyrotropin-sensitive immunoglobins bind to thyrotropin receptors in periorbital tissue, causing an inflammatory response that includes invasion of T-lymphocytes and macrophages. This creates fibroblasts between muscle fibres and orbital fat that become oedematous, with local cytokines magnifying the inflammatory response.^1,2,5,7,8^ Radiotherapy is effective in suppressing inflammation and inhibiting lymphocyte infiltration.^5,7,8^ The natural history of TED can vary widely from spontaneous remission to increasing severity, with three phases (active, inactive and burnout) determined by a clinical activity score (CAS).^1,3^ OR is reserved for active disease; inactive disease management includes symptom alleviation using artificial tears, sunglasses or prisms.^7^ Orbital decompression to relieve proptosis, muscle corrective surgery for recti muscles and eyelid oculoplastic surgery can be performed once the condition becomes inactive.^5^ Somatostatin analogues are another systemic option that could be used in combination with steroids.^9^ TED is difficult to treat because of the variability of symptoms expressed, length of disease phases, and factors such as thyroid status and smoking, which may alter  its natural history.^10^

Radiotherapy remains controversial owing to inconsistent results from previous trials, especially with proptosis and diplopia. There is also a lack of data on the effectiveness of OR with corticosteroids, as most prospective studies are OR *vs* sham irradiation or corticosteroids.^2,7,8,11^ An OR *vs* sham study only showed significant changes in motility (82% OR *vs* 27% sham) in moderate and severe cases, without significant improvements in proptosis or eye swelling.^7^ However, another study showed an improvement in proptosis (52% *vs* 27%) for mild cases. These results could have been confounded by treating mild TED at its inactive phase, which would underestimate its therapeutic ability.^8^ Additionally, the two trials grouped outcome measures into “major” and “minor” changes, which may also over- or underestimate the improvement of each symptom.^7,8^ Marquez et al^2^ utilized post-OR patient questionnaires after 1 year and ophthalmologic evaluations scored by SPECS (periobital soft tissue changes, proptosis, extraocular muscle dysfunction, corneal abnormalities and sight changes) to evaluate efficacy. 84% of patients responded with an improvement in symptoms, and 89% of SPECS scores were lowered.^2^ Almost all studies agree that OR is effective in treating visual acuity and optic nerve compression.^2,3,5,11^ Hahn et al^3^ have shown combination therapy to be effective, improving diplopia (31%), proptosis (2.5 mm left and 2.0 mm right), visual acuity (81%) and extraocular movement (58%). OR was evaluated retrospectively by the ability to taper from corticosteroids: 90.9% of patients were off it within 6 months without further exacerbation.

Variations in treatment delivery [energy, beam design, source-axis distance (SAD) and fractionation] may have affected treatment results. One study involved irradiation of one eye using a wedge pair and sham with the contralateral over 6 months and *vice versa*.^11^ However, the sham-irradiated eye received a dose of 2 Gy, which was enough to cause therapeutic changes.^5^ Angling beams posteriorly to avoid the lenses compared with half-beam blocking could affect dose distribution. Recently, several studies on using advanced radiotherapy modalities such as intensity modulated radiation therapy and tomotherapy have shown better dose uniformity and sparing to normal tissues.^12,13^ Almost all studies used isocentric positioning except for one that had a SAD of 128.3 cm.^2,7,8,11^ 20 Gy in 10 fractions was most widely used in the literature reviewed, although other fractionation schemes (1 Gy per week for 10 or 20 weeks) have shown clinically significant therapeutic responses.^14,15^ Almost all studies agree on the tolerability and safety of OR, showing no increase in secondary malignancies and difference in survival.^2,4,5^ Marcocci et al^4^ showed cataract formation in 12% of patients with a median of 11 years after treatment and a median age of 59 years.

OR with corticosteroids has been shown to be effective and tolerable, improving our patient’s visual acuity and colour vision, and reducing upper lid oedema. Our patient benefited from active monitoring that led to quick intervention during its active phase. However, she may require further intervention after an extended period of disease inactivity. Prospective studies of OR and steroid combination therapy with consistent parameters (CAS score and ocular symptoms) and patient eligibility (disease activity and smoking status) are needed to provide proper analyses of its effectiveness.

## Learning Points

1. TED is rarely seen in the radiotherapy setting. We see the unique aspects of this disease and the multidisciplinary approach needed in this study.
2. This case report provides an overview of how this treatment was planned and delivered, and highlights new techniques that could be implemented.
3. TED requires timely intervention owing to the unpredictable nature of disease progression.
4. Further studies on the use of radiotherapy in a controlled environment is needed to understand the true efficacy and mechanisms of said intervention.

## Consent

Patient had given consent to the use of images and medical records.
